# Tongue Osseous Choristoma in an Adolescent Female: A Case Report

**DOI:** 10.7759/cureus.32275

**Published:** 2022-12-06

**Authors:** Nasser Almujaiwel, Kawther Alabbas, Waleed Alshareef, Nasser Almutairi, Latifa AlMakoshi

**Affiliations:** 1 Department of Otolaryngology - Head and Neck Surgery, King Saud University Medical City, Riyadh, SAU; 2 Department of Pathology, King Khalid University Hospital, Riyadh, SAU; 3 Department of Otolaryngology - Head and Neck Surgery, King Faisal Specialist Hospital, Riyadh, SAU

**Keywords:** pedunculated mass, tongue mass, osteoma mucosae, lingual osteoma, osseous choristoma

## Abstract

Lingual osseous choristoma is a rare, benign, bony tumor without clear pathogenesis. Most patients present with an asymptomatic lump in the posterior tongue, while others may suffer from globus sensation, dysphagia, gagging, or irritation. Here, we present a case of lingual osseous choristoma in a pediatric patient managed with surgical excision.

## Introduction

Choristoma is a developmental tumor-like growth of microscopically normal tissue in an abnormal location. Osseous choristoma is a benign bony tumor that consists of normal mature osseous tissue occurring in the soft tissue of either the skin (osteoma cutis) or the mucosa of the oral cavity (osteoma mucosae) [1,2]. The lesion tends to develop in the head and neck region, mainly in the posterior region of the tongue. Choristoma has been reported to be found in the middle ear as well [3]. Monserrat reported the first case of osseous lesion in the tongue in the year 1913 and used the term “lingual osteoma,” which is a benign neoplasm of bone [4]. Because of its rarity, we are presenting this case of a choristoma located in the posterior region of the tongue in an adolescent patient with a brief review of the literature on tongue osseous choristoma.

## Case presentation

**Figure 1:**
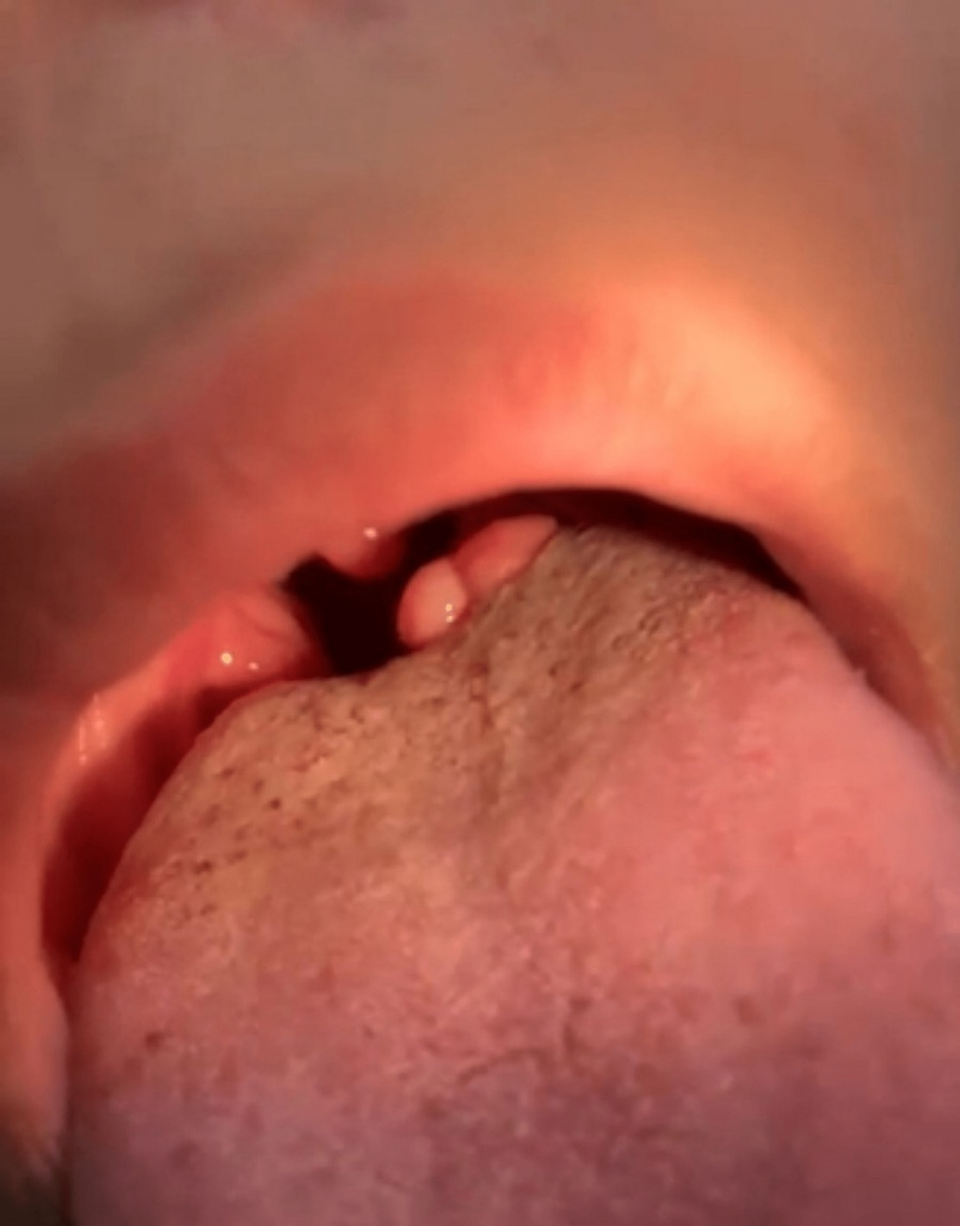
A pedunculated nodule in the left posterior side of the tongue.

**Figure 2:**
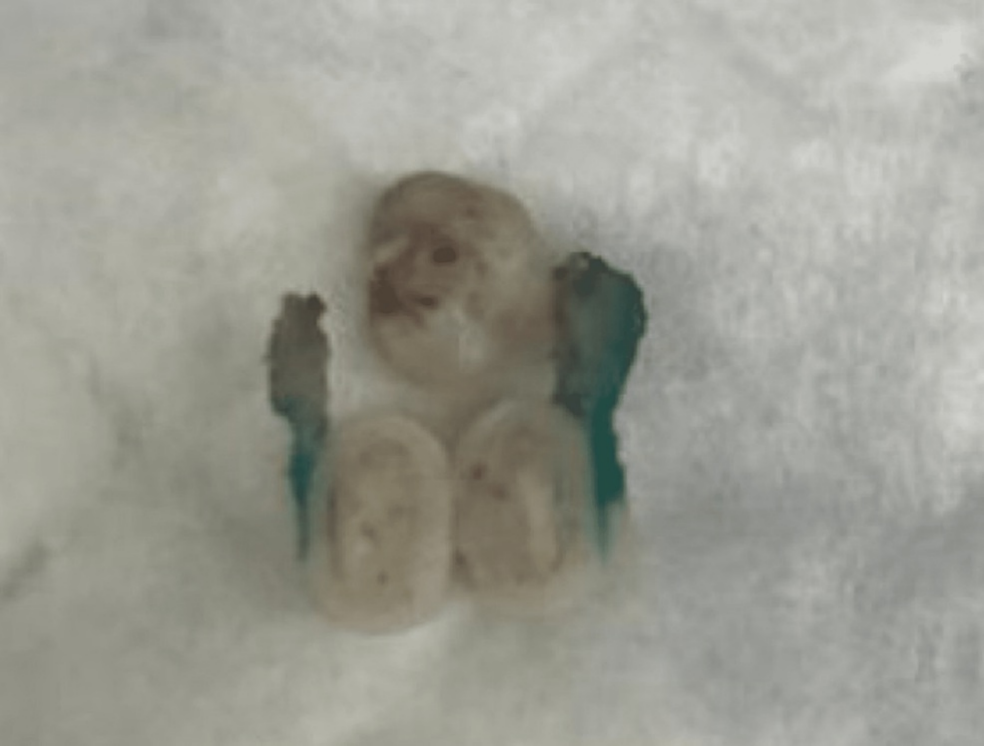
Gross picture of a round white bony lesion taken from the posterior side of the tongue. The cut surface is densely sclerotic.

**Figure 3:**
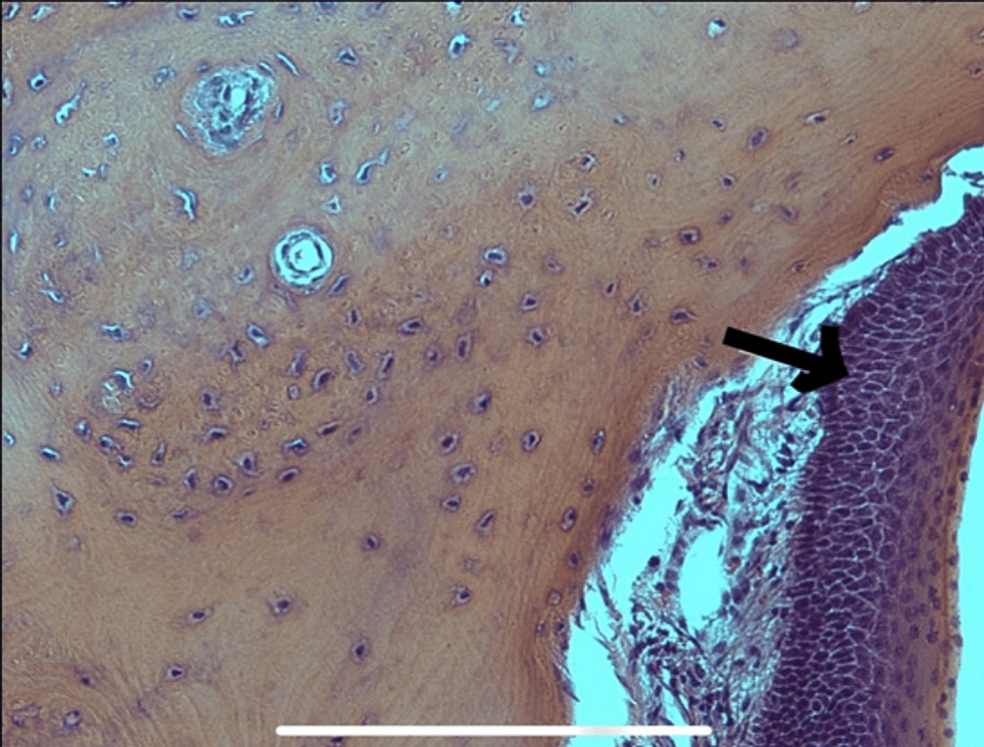
Squamous epithelium (arrow) with underlying mature lamellar bone with Haversian-like canal rimmed with osteoblast.

**Figure 4:**
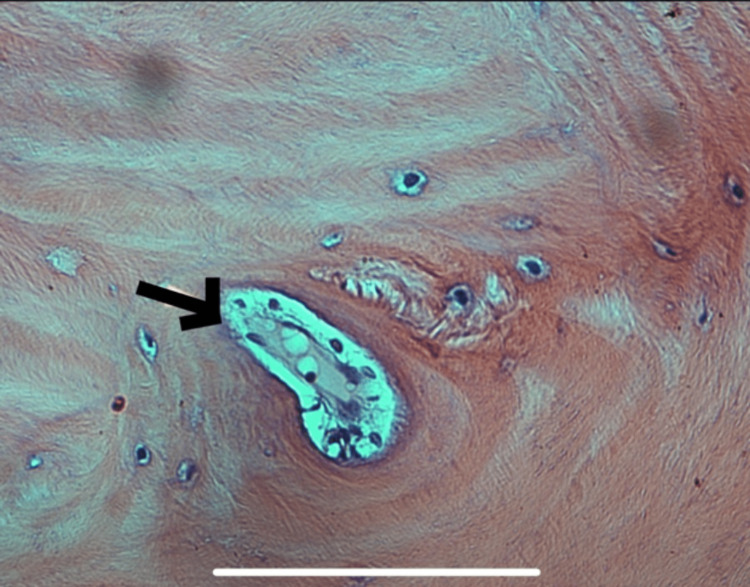
High power view of mature lamellar bone with Haversian-like canal (arrow) rimmed with osteoblast.

A 12-year-old healthy girl was referred to our pediatric otolaryngology clinic for assessment of a tongue mass. She was complaining of a mass over the left posterior side of her tongue for two years. The mass was generally stable in size, with slight swelling noticed during upper respiratory tract infection attacks. The child felt some heaviness while talking or eating. The family also reported associated snoring and change in voice. Oral examination showed a 1.7 cm x 1 cm pedunculated, firm, non-tender mass with a smooth surface in the left posterior dorsum of the tongue (Figure 1). No other lesions or neck swelling were noticed, and fiberoptic nasolaryngoscopy was unremarkable. Due to its benign appearance, ultrasound and cross-sectional imaging were not requested. An excisional biopsy was performed, and Molt mouth gag was used as a prop. The histopathology result came out as osseous choristoma. Gross and microscopic examination of the lesion is described in Figures 2-4. The postoperative period was uneventful, and the patient reported improvement in symptoms with no recurrence during one year of follow-up.

## Discussion

Osseous choristoma of the tongue is an extremely rare condition, and its exact cause is unknown. It has been estimated that less than 100 cases have been reported since Monserrat (1913). The other term used for this condition is lingual osteoma, which is not accurate, as osteoma is a benign, progressive neoplasm arising from osteogenic tissue and associated with the bones. Applying this condition to lingual osteoma, no relationship between the lesion and the bones or any proven osteogenic origin could be observed. Also, not all lingual lesions tend to enlarge [4,5]. Osseous choristoma arises almost three times more commonly in females than in males. The patients’ age ranged from 5 to 73 years, with a mean of 28.7 years, and the majority of cases occurred in the second or third decades of life [6]. Furthermore, regarding pediatric cases, Arimoto et al. reviewed the literature and found 17 cases, including 13 of them being females. No identifiable reason for the sexual predisposition and no major differences in clinicopathological characteristics between pediatrics and adults were observed [7]. The most frequently affected region is the posterior third of the tongue dorsum near the foramen caecum and circumvallate papillae accounting for 90% of cases, and 10% are on the middle third of the tongue [8]. In terms of clinical presentation, most of the patients are asymptomatic, and the most frequent symptom is a lump (46%), followed by dysphagia (6.9%), gagging (5.1%), nausea (3.4%), and irritation (3.4%) [9,10]. It appears as a sessile or pedunculated mass that varies from 3 mm to 50 mm in size. The differential clinical diagnosis can be based on the tumor location. On the dorsal tongue near the foramen caecum, we should consider benign tumors like hemangioma, lymphangioma, teratoma, hamartoma, and leiomyoma; other differentials are thyroglossal duct cyst, lingual thyroid, mucocele, and pyogenic granuloma, and malignant tumors such as rhabdomyosarcoma and epidermoid carcinoma should also be kept in mind. As for the lateral side of the tongue, it includes traumatic neuroma, neurofibroma, schwannoma, fibroma, and cartilaginous choristoma. Pyogenic granuloma, mucocele, and cartilaginous choristoma usually involve the anterior part of the tongue [9]. A definitive diagnosis was made by histopathologic examination. The osseous choristoma resembles the normal lamellar bone elsewhere in the body. While the Haversian systems, osteocytes, and fatty bone marrow were present, osteoblast and osteoclast were rarely seen [8]. Management was done with surgical excision. Recurrent cases of intraoral osseous choristoma have been reported, but none of them involved the tongue. No cases of malignant transformation have been reported [10,11]. Our case is the 18th one reported in the literature of tongue osseous choristoma in a pediatric population with a similar clinical presentation.

The pathogenesis of osseous choristoma is still unknown, but multiple theories have been discussed in the literature. The first theory was by Monserrat [4], who suggested that it is derived from the branchial arch as a developmental malformation. The union between the anterior two-thirds of the tongue originates from the first branchial arch, and its posterior third originates from the third branchial arch in the region of the foramen caecum and the sulcus terminalis. It is known that certain normal bony structures are derived from these branchial arches, such as the incus and malleus from the first arch and most of the hyoid bone from the third arch. Therefore, ossified branchial arch remnants and subsequent development of an osseous lesion in the tongue are possible. This theory is supported by the location of choristoma [12]. The second theory is on remnant ossified thyroid tissue, which originates at the foramen caecum and descends into its normal position. But so far, no thyroid tissue has been found in current specimens to support this theory [13]. The post-traumatic reaction is the third theory explaining this condition, as it indicates that the posterior third of the tongue is susceptible to irritation due to trauma, swallowing, and articulation. Chronic inflammatory cells in the epithelium and in the connective tissue are a common finding in these types of lesions with subsequent calcification; this process is also seen in skeletal muscles known as “myositis ossificans,” a condition representing ossification inside a muscle after trauma; the latter usually lacks the Haversian system, which is opposite to osseous choristoma [14,15].

## Conclusions

Tongue osseous choristoma is an extremely rare benign bony lesion occurring mostly in the posterior side of the tongue. It tends to develop more in females and most patients are asymptomatic. Surgical excision is the diagnostic and treatment modality. Reporting such a case will increase awareness of the disease in the otolaryngology community.
